# Intraluminal Migration of Surgical Mesh With Retained Metallic Tacks Causing Small Bowel Obstruction After Ventral Hernia Repair: A Case Report

**DOI:** 10.7759/cureus.113781

**Published:** 2026-08-01

**Authors:** Abenezer S Tedla, Katherine Rivero, Ariana Figueroa, Frank J Masella, Daniel T Farkas

**Affiliations:** 1 General Surgery, BronxCare Health System, New York, USA; 2 Surgery, BronxCare Health System, New York, USA; 3 Surgery, School of Medicine, Ross University School of Medicine, New York, USA; 4 Surgery, Bronx-Lebanon Hospital Center, New York, USA

**Keywords:** case report, mesh migration, metallic tacks, small bowel obstruction, ventral hernia repair

## Abstract

Mesh repair is commonly used for abdominal wall hernias. However, mesh-related complications, including infection, adhesion formation, and migration, remain important considerations. Mesh migration into the gastrointestinal tract represents an uncommon delayed postoperative complication that can manifest long after the initial surgical repair. Patients may experience a range of gastrointestinal symptoms depending on the location and extent of migration. We report a case of small bowel obstruction caused by intraluminal migration of surgical mesh with retained metallic fixation tacks.

A 60-year-old male with a history of prior abdominal surgeries, including abdominoplasty and laparoscopic surgery for bowel obstruction approximately 15 years earlier, presented with worsening colicky abdominal pain, nausea, and vomiting. A CT of the abdomen and pelvis demonstrated a mechanical small bowel obstruction with a heterogeneous intraluminal density in the distal ileum. Exploratory laparotomy revealed dense adhesions and a firm intraluminal foreign body measuring approximately 7 cm within the distal ileum. The foreign body was identified as folded surgical mesh with embedded metallic fixation tacks that had migrated intraluminally. The affected bowel segment was resected, and a stapled anastomosis was performed. The postoperative course was complicated by prolonged ventilatory support and intra-abdominal abscesses requiring percutaneous drainage, after which the patient improved clinically.

Intraluminal mesh migration is an uncommon but clinically important cause of small bowel obstruction that should be considered in patients with prior hernia repair presenting with obstructive symptoms, particularly when imaging demonstrates an intraluminal foreign body. Surgical management is often necessary and may carry significant morbidity due to dense adhesions and the need for bowel resection. A high index of clinical suspicion is essential for timely diagnosis and appropriate operative planning.

## Introduction

Abdominal wall hernia repair is one of the most commonly performed procedures in general surgery, with approximately one million meshes used annually worldwide for hernia repairs [1]. The use of prosthetic mesh has become the standard of care in abdominal wall hernia repair due to reduced recurrence rates [2,3]. However, mesh-related complications can occur, including infection, adhesion formation, erosion, and migration [4,5]. 

Mesh migration into the gastrointestinal tract, specifically the cecum, small bowel, transverse colon, and sigmoid colon, is a rare but recognized complication that may occur years after the initial surgery [6,7]. In a comprehensive review by Cunningham et al., 84 articles describing mesh migration following abdominal hernia repair were identified, with the index hernia repair being incisional or ventral in 28.1% of cases and migration commonly affecting multiple organs (31.5%) [7]. Two principal mechanisms of mesh migration have been described: primary mechanical migration due to inadequate fixation and secondary migration due to chronic inflammatory erosion into adjacent viscera [5,6]. Koliakos et al. reported that late-presenting intestinal erosions (>6 months postoperatively) were significantly more likely to involve mesh erosion and to require bowel resection compared to early-presenting cases [6]. 

Clinical presentations vary and may include bowel obstruction, perforation, or fistula formation [5,6]. Acute obstruction is the most common presenting symptom, occurring in 31.5% of reported cases of intestinal erosion following hernia repair [6]. Millas et al. described a case of chronic abdominal pain resulting from mesh migration and erosion into the sigmoid colon from a distant supraumbilical hernia repair site, illustrating that migration can occur across considerable anatomical distances [8]. A CT is the primary imaging modality for evaluating suspected mesh complications, though mesh visibility at imaging is variable depending on mesh composition and the nature of the complication [4].

The role of metallic fixation devices in mesh-related complications has also been highlighted; displaced spiral tacks used for mesh or peritoneal fixation have been independently reported as causes of small bowel obstruction and perforation [9,10]. In contrast, intraluminal migration of prosthetic mesh with retained metallic fixation tacks acting together as a discrete intraluminal foreign body represents a particularly unusual manifestation that should be distinguished from simple mesh erosion into the bowel wall or isolated tack migration. To our knowledge, this presentation remains exceedingly rare, particularly involving the distal ileum. Norton et al. described a case of intraluminal mesh migration into the transverse colon and distal ileum after ventral hernia repair, emphasizing the need for a high index of suspicion [11]. This entity may closely mimic more common causes of small bowel obstruction on both clinical presentation and imaging, particularly when CT findings are subtle or nonspecific. Consequently, establishing the diagnosis requires a high index of suspicion and careful correlation of imaging, operative findings, and prior surgical history.

We present a case of distal ileal obstruction caused by migrated surgical mesh approximately a decade after presumed ventral hernia repair. This case highlights an uncommon mechanism of small bowel obstruction in which migrated mesh with retained tacks masqueraded as more common etiologies, emphasizing the importance of maintaining clinical suspicion for late mesh-related complications even when prior operative history is limited and preoperative imaging is inconclusive.

## Case presentation

A 60-year-old man with a past medical history significant for obesity and a surgical history of abdominoplasty and a reported laparoscopic management of bowel obstruction secondary to a ventral hernia approximately 15 years earlier presented with a one-month history of intermittent colicky abdominal pain. His symptoms acutely worsened over three days and were associated with nausea and multiple episodes of vomiting. 

On presentation, vital signs were within normal limits: blood pressure 140/75 mmHg, heart rate 70 beats per minute, temperature 37.1°C, respiratory rate 17 breaths per minute, and oxygen saturation 99% on room air. Physical examination revealed multiple well-healed surgical scars, including lower transverse and laparoscopic port scars. The abdomen was slightly distended and tender in the right lower quadrant without rebound tenderness, guarding, or signs of peritonitis. Laboratory evaluation was notable for leukocytosis (WBC 13.9 × 10³/µL; normal range: 4-11× 10³/µL) with neutrophilic predominance (81.1%), anemia (hemoglobin 8.5 g/dL; normal range: 12-16 g/dL), and a normal lactic acid level (1.4 mmol/L; normal range 0.5-2.0 mmol/L). The leukocytosis with neutrophilia was consistent with an acute inflammatory or obstructive process, while the normal lactate was reassuring against bowel ischemia (Table 1). 

**Table 1:** Laboratory evaluation at presentation

Laboratory parameter	Patient value	Reference range	Units	Interpretation
WBC count	13.9	4.0-11.0	×10³/µL	Leukocytosis suggestive of an acute inflammatory response
Neutrophils	81.1	40-70	%	Neutrophilic predominance consistent with acute inflammatory process
Hemoglobin	8.5	12-16	g/dL	Anemia identified on presentation
Serum lactate	1.4	0.5-2.0	mmol/L	Normal level, reducing concern for advanced bowel ischemia

**Figure 1:**
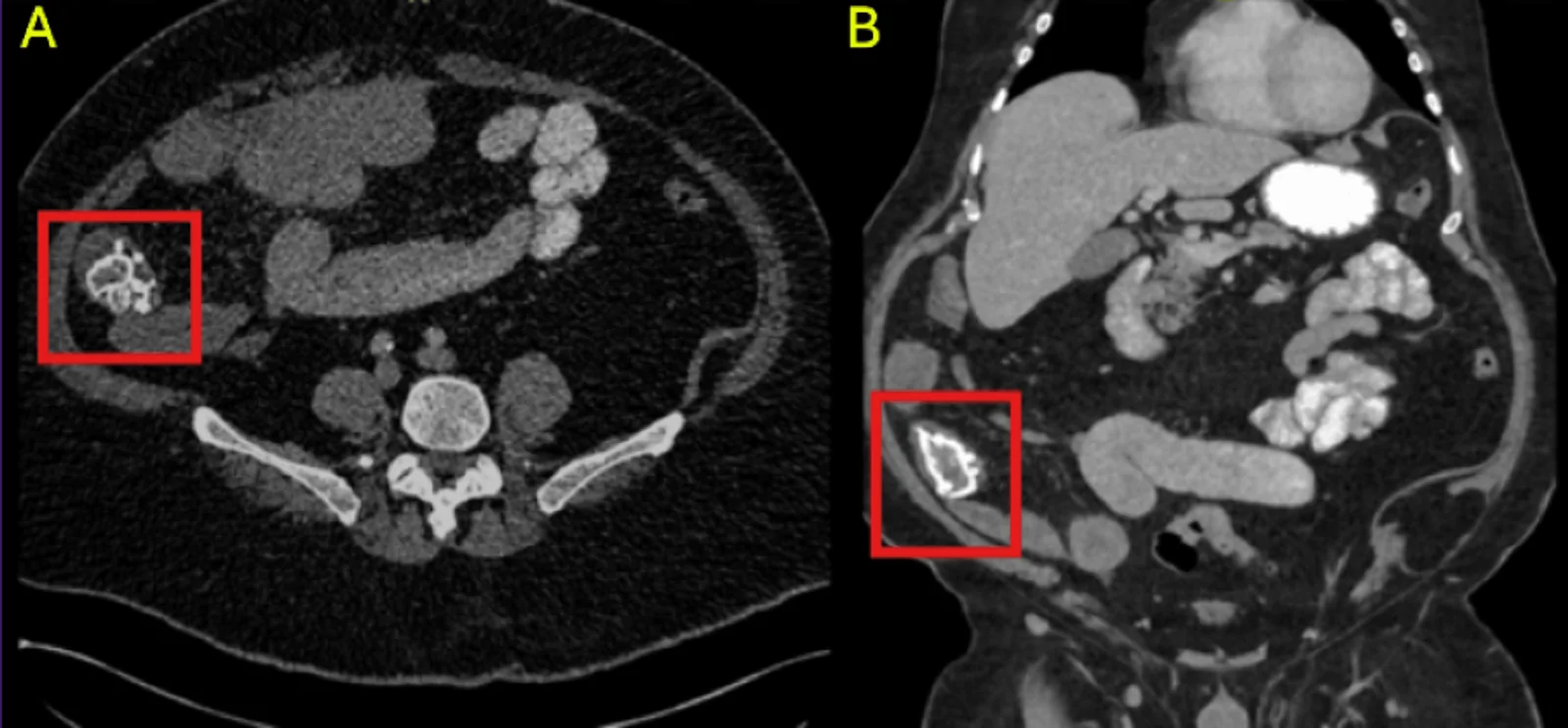
CT of the abdomen and pelvis demonstrated a small bowel obstruction with an intraluminal foreign body in the distal ileum A: Axial view, B: Coronal view

A contrast-enhanced CT scan of the abdomen and pelvis demonstrated a 4 cm × 3.5 cm heterogeneous intraluminal density within the distal small bowel in the right lower quadrant, with associated upstream small bowel dilation, decompressed distal bowel, and adjacent mesenteric inflammatory changes, consistent with a mechanical small bowel obstruction (Figure 1).

**Figure 2:**
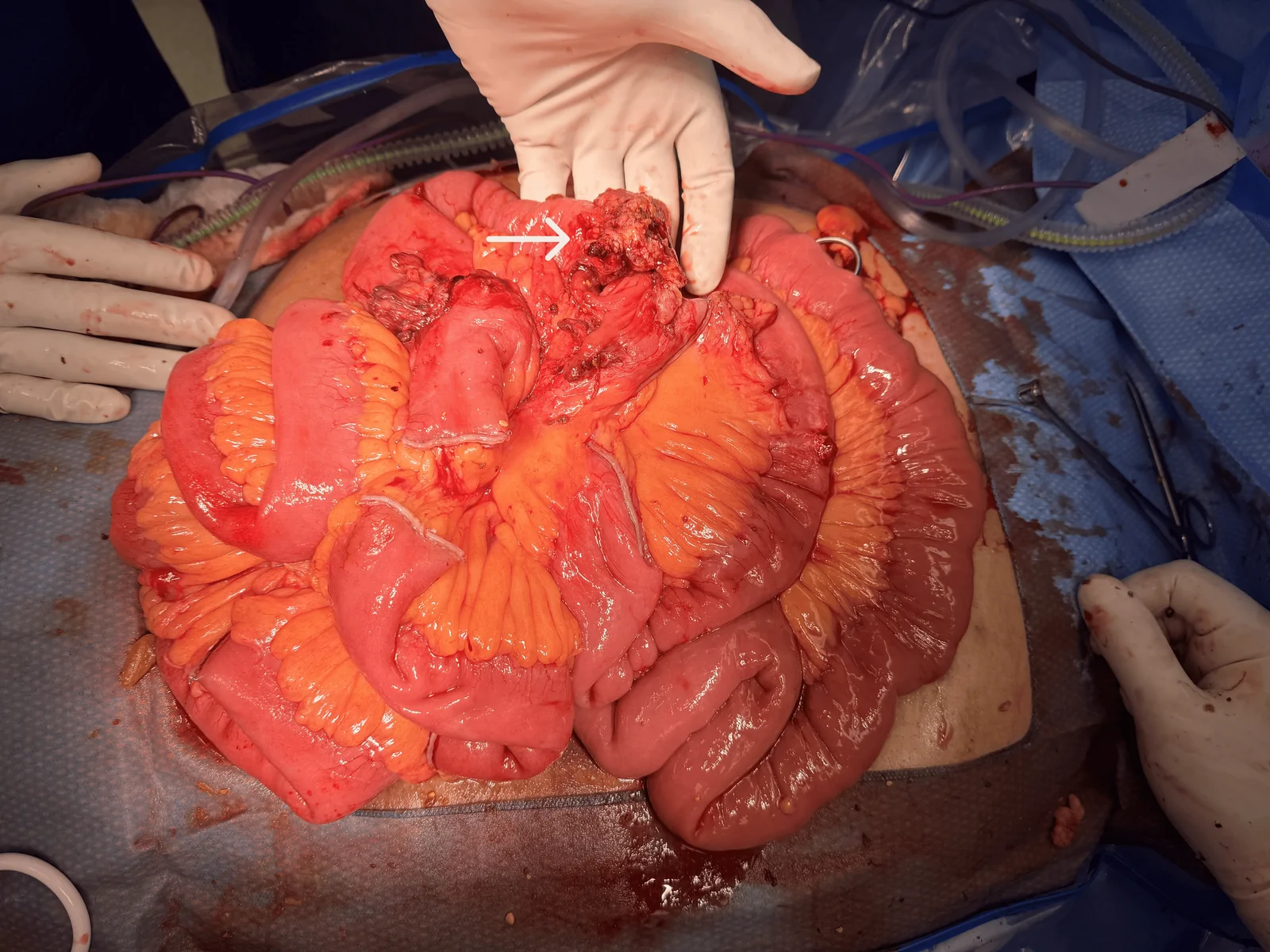
Small bowel segment adherent to the abdominal wall corresponding to the site of mesh transmural migration (white arrow)

Given the presence of an intraluminal foreign body causing small bowel obstruction, the patient was taken to the operating room for exploratory laparotomy on the day of presentation. Upon entry via a midline incision, tight, dense adhesions were encountered between the small bowel and the anterior abdominal wall. Enterotomies were unavoidable during adhesiolysis of the small bowel off the abdominal wall, resulting in significant enteric spillage. A Prolene suture was noted embedded within the bowel involved with these dense adhesions. There was an area of small bowel segment adherent to the abdominal wall corresponding to the site of transmural migration (Figure 2).

**Figure 3:**
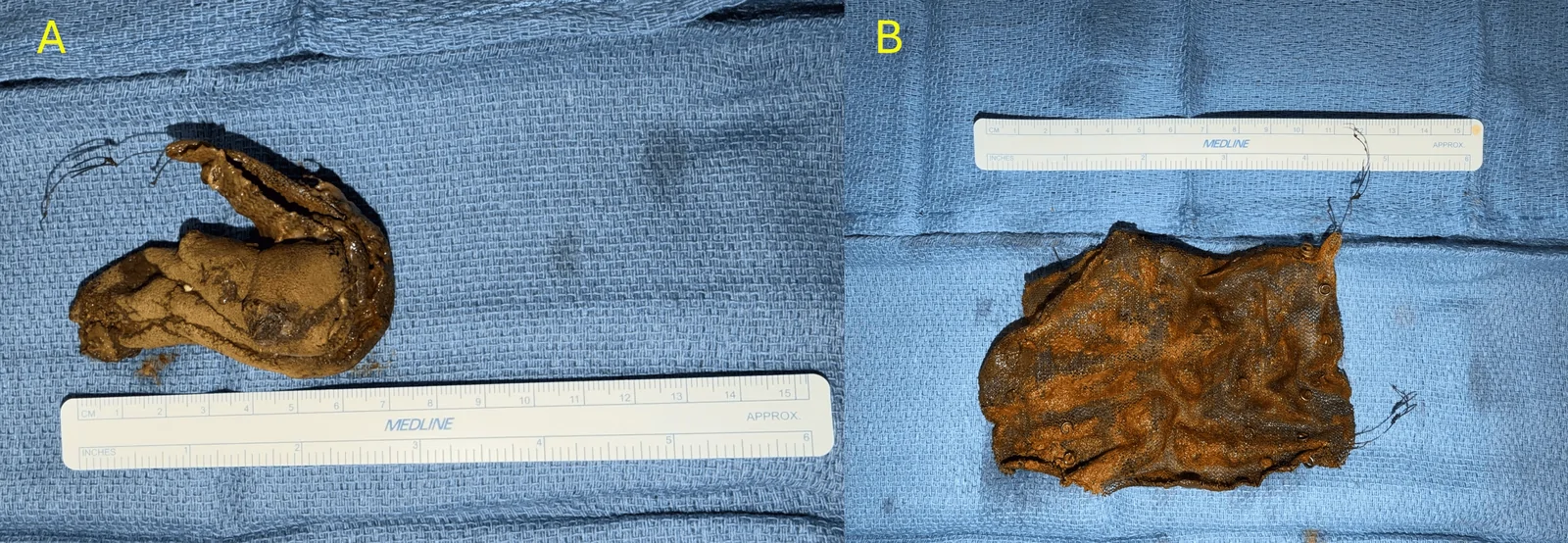
Gross examination images of the mesh with metallic tacks and sutures visible A: Folded mesh, B: Unfolded mesh

Further inspection revealed a segment of distal ileum containing a firm, elongated intraluminal foreign body measuring approximately 7 cm. The object was carefully milked proximally and extracted through the distal enterotomy. Gross examination revealed a folded surgical mesh with embedded tacks and positioning sutures attached at three corners (Figure 3)

A segmental bowel resection was done, removing all the inflamed bowel and enterotomies as one segment. A single stapled bowel anastomosis was done, and the mesenteric defect was closed. The remaining bowel was inspected and found to be viable. The patient required transfusion of two units of packed RBCs intraoperatively due to hemodynamic lability but responded appropriately to resuscitation and was transferred to the intensive care unit. 

Additional history obtained after surgery revealed that a CT scan performed during an admission 15 years earlier at an outside hospital demonstrated bowel obstruction secondary to incarceration of bowel within an umbilical hernia. We suspect that the umbilical hernia was subsequently reduced and repaired with mesh placement at the defect; however, the official operative report was unavailable for review.

**Figure 4:**
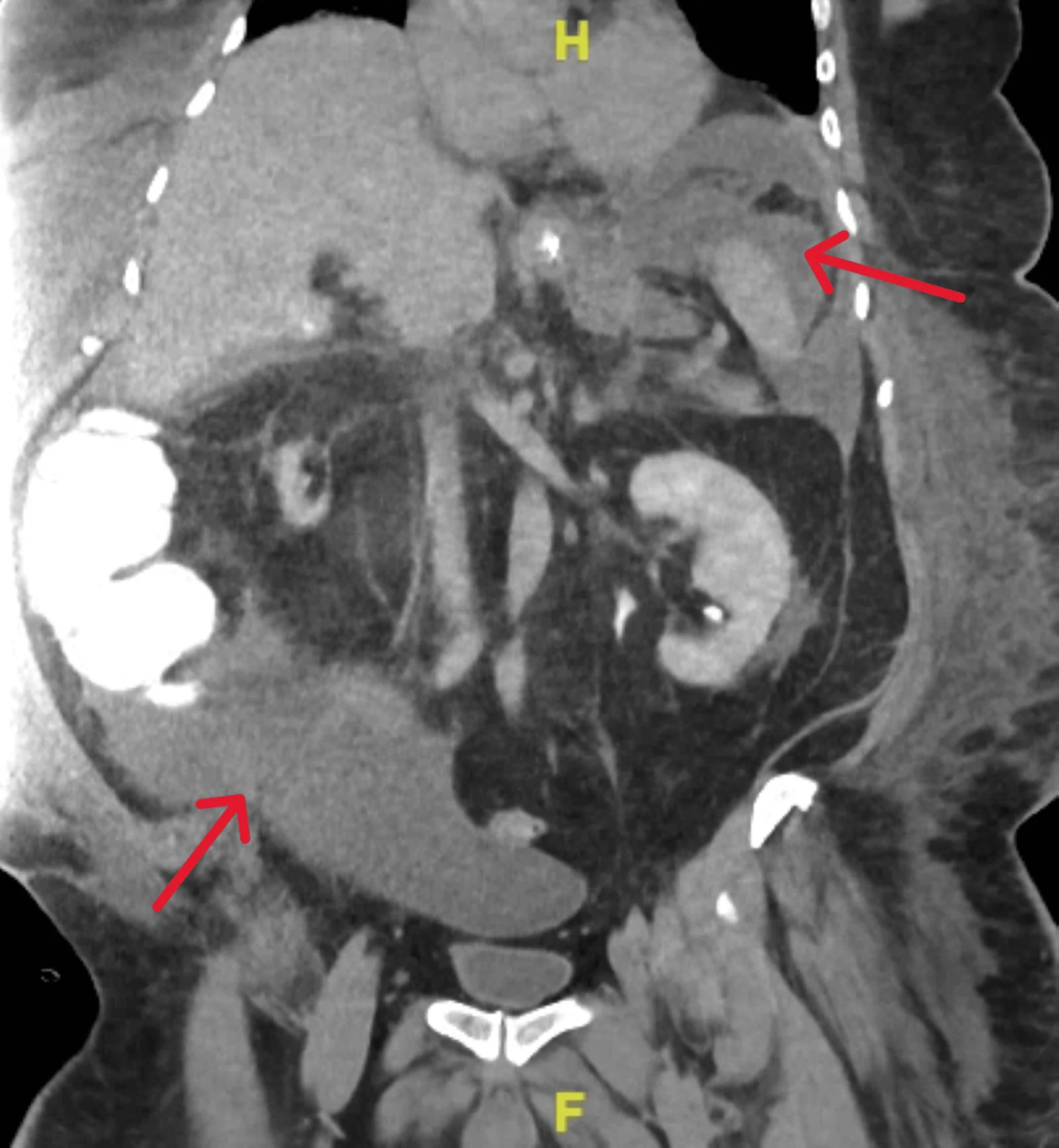
Coronal view of the CT demonstrated intra-abdominal fluid collections (red arrows)

Postoperatively, the patient required prolonged ventilator support and was extubated on postoperative day seven. His course was further complicated by the development of multiple intra-abdominal collections, including perisplenic and pelvic abscesses, which were successfully managed with interventional radiology-guided percutaneous drainage (Figure 4). The patient subsequently improved clinically, was discharged in stable condition, and was doing well at follow-up.

## Discussion

Mesh migration is a rare but well-documented late complication of prosthetic hernia repair, with presentations reported years to decades after the initial procedure [5-7]. In a comprehensive review of 84 articles, Cunningham et al. found that the average age of affected patients was 59.8 ± 13.8 years with a male predominance (76.2%), and the index hernia repair was incisional or ventral in 28.1% of cases [7]. Two principal mechanisms have been described: primary mechanical migration due to inadequate fixation or external forces, and secondary migration resulting from chronic inflammation leading to gradual erosion into adjacent viscera [5,6]. Cases that present after six months were significantly more likely to involve mesh-erosion rather than fixation-material-erosion alone [6]. 

In this case, intraoperative findings strongly support a mechanism of chronic erosion, but inadequate fixation also is suspected. Dense adhesions between the small bowel and anterior abdominal wall, along with the presence of a Prolene suture embedded within these adhesions, suggest chronic inflammation at the site of prior mesh placement. However, the fact is that despite metal tacks being used, the entire mesh with the tacks came free from the abdominal wall, aside from one remaining tack. This points to inadequate fixation. Given the additional history obtained after surgery, with strangulated bowel within the hernia, we suspect that there was some contamination of the mesh at the time of placement. We did not see evidence of a bowel resection, but even if the bowel was viable, it’s possible or even likely that there was bacterial translocation within the bowel in the hernia sac. This could have led to some level of infection at the time of placement, which may have led to inadequate incorporation of the mesh to the abdominal wall. 

Migration into the small bowel is relatively uncommon compared to involvement of the colon or urinary bladder [5,7]. When present, it may manifest as obstruction, perforation, fistula formation, or chronic abdominal pain [4]. Gallagher et al. described a case of small bowel obstruction caused by bezoar formation around intraluminal hernia mesh, illustrating the diverse mechanisms by which migrated mesh can cause obstruction [12]. In our patient, the mesh migrated intraluminally and functioned as a mechanically obstructing foreign body within the distal ileum. The role of metallic tacks in mesh migration warrants specific consideration. While tack fixation is widely used in laparoscopic ventral hernia repair and has been shown to have comparable outcomes to suture fixation in terms of recurrence and chronic pain, displaced tacks have been reported to cause small bowel obstruction and perforation [2,13,14]. In our case, the metallic tacks remained embedded within the migrated mesh, contributing to the formation of a discrete intraluminal foreign body. 

Several risk factors have been identified. Patient-level risk factors include female sex, younger age, emergency repair, obesity, immunosuppressive therapy, and surgical site infection, all of which increase the risk of mesh-related complications including infection, foreign body reaction, and subsequent migration [15,16]. Mesh position also plays a role in visceral complications. Intraperitoneal placement allows direct visceral contact and is associated with the highest risk of adhesion formation and bowel obstruction [17]. Among surgical techniques, laparoscopic inguinal hernia repair (TAPP/TEP) has the highest reported incidence of mesh-related visceral complications, whereas the Lichtenstein technique has the lowest [5,6].

Preoperative diagnosis remains challenging. Imaging with CT is the modality of choice in the evaluation of small bowel obstruction and may reveal a transition point with an associated intraluminal foreign body. However, distinguishing migrated mesh from other intraluminal contents can be difficult, particularly when prior surgical history is unclear. Gavlin et al. noted that mesh visibility on CT is variable, and awareness of the patient's surgical history is essential for accurate interpretation [4]. Definitive management is surgical [6,7]. Although minimally invasive approaches may be considered, the presence of extensive adhesions, bowel injury, or contamination can influence the decision to proceed with open exploration. In the systematic review by Koliakos et al., bowel resection was required in 84.1% of late-presenting cases [6]. The Eastern Association for the Surgery of Trauma guidelines recommend timely surgical exploration for patients with small bowel obstruction and evidence of clinical deterioration, including leukocytosis [18]. 

Given this patient's history of ventral hernia repair with mesh following bowel obstruction due to an incarcerated umbilical hernia, one possible mechanism for the delayed mesh migration is impaired mesh incorporation resulting from subclinical contamination at the time of the index operation. The presence of incarcerated or potentially strangulated bowel raises concern for bacterial translocation, which may have led to occult contamination of the surgical field despite the absence of overt infection. Such contamination could have interfered with normal tissue ingrowth and mesh integration, predisposing the prosthesis to gradual displacement and eventual intraluminal migration. Although this mechanism cannot be confirmed in the present case, it represents a biologically plausible explanation supported by the clinical context.

This case also underscores the potential for significant morbidity. Dense adhesions led to unavoidable enterotomies and intra-abdominal contamination, contributing to a complicated postoperative course requiring prolonged ventilatory support and image-guided drainage of intra-abdominal collections. Kokotovic et al.'s large cohort study reported that mesh-related complications following incisional hernia repair included bowel perforation due to mesh migration and bowel obstruction, with three deaths attributable to mesh-related complications [3]. This case emphasizes the importance of considering migrated mesh in the differential diagnosis of small bowel obstruction in patients with prior abdominal wall repair, particularly when imaging suggests an intraluminal foreign body. 

## Conclusions

Intraluminal migration of prosthetic mesh is an uncommon but important late complication of abdominal wall hernia repair that may present years after the initial operation and should be considered in patients with a history of prior mesh placement who develop symptoms of bowel obstruction. This case highlights the diagnostic value of correlating cross-sectional imaging findings with a patient’s surgical history and demonstrates that mesh migration with retained metallic fixation tacks can serve as an intraluminal foreign body causing mechanical obstruction. Definitive management typically requires operative intervention with mesh explantation and resection of the affected bowel segment when necessary. Awareness of this potential complication may facilitate earlier diagnosis, appropriate surgical planning, and improved patient outcomes.
